# Case Report: Eosinophilic Myenteric Ganglionitis in a Child With Hirschsprung's Disease: A Challenge in Pseudo-Obstruction

**DOI:** 10.3389/fped.2020.617309

**Published:** 2021-02-04

**Authors:** Enza D'Auria, Valeria Calcaterra, Luciano Maestri, Milena Meroni, Giorgio Giuseppe Orlando Selvaggio, Vincenzo Villanacci, Manuela Nebuloni, Gloria Pelizzo

**Affiliations:** ^1^Allergology Unit, Department of Pediatrics, “Vittore Buzzi” Children's Hospital, Milan, Italy; ^2^Department of Biomedical and Clinical Science “L. Sacco”, University of Milan, Milan, Italy; ^3^Pediatrics and Adolescentology Unit, Department of Internal Medicine, University of Pavia, Pavia, Italy; ^4^Department of Pediatrics, “Vittore Buzzi” Children's Hospital, Milan, Italy; ^5^Pediatric Surgery Unit, Vittore Buzzi Children's Hospital, Milan, Italy; ^6^Institute of Pathology, Spedali Civili, Brescia, Italy; ^7^Pathology Unit, Department of Biomedical and Clinical Sciences, L. Sacco Hospital, University of Milan, Milan, Italy

**Keywords:** eosinophilic myenteric ganglionitis, children, Hirschsprung's disease, pseudo- obstruction, diagnosis, endoscopy

## Abstract

**Introduction:** The presentation of eosinophilic myenteric ganglionitis (EMG) can be similar to that of Hirschsprung's disease (HD). In a limited number of cases of pediatric patients, the diagnosis of both EMG and HD are reported. A case of pseudo-obstruction in EMG occurring in a child with HD diagnosis is discussed with literature review.

**Case Presentation:** A boy aged 2 years and 6 months presented with intractable constipation and abdominal distension. Histological HD diagnosis was carried out and transanal Soave pullthrough was performed. At the age of 3 years and 2 months, an infectious enterocolitis occurred. One month later, he presented with constipation, marked abdominal distension and melena. Full thickness colonic biopsies revealed eosinophilic myenteric ganglionitis. Specific IgE tests were positive for several foods. Dietary exclusion was adopted with resolution of clinical symptoms and histologic remission.

**Conclusion:** EMD may occur in patients with HD. At the onset, EMD may be associated with functional intestinal obstruction. The use of an elimination diet proved effective for the relief of symptoms. Long term follow-up is mandatory to define the timing of the reintroduction of foods.

## Introduction

Eosinophilic myenteric ganglionitis (EMG) is an inflammatory neuronal enteropathy, characterized by infiltration of the Auerbach myenteric plexus by eosinophils, which develops mainly in children (1). EMG can be associated with bowel dysmotility and has rarely been reported as a cause of intestinal pseudo-obstructive syndrome (1).

Hirschprung's disease (HD) is the most common cause of congenital intestinal dysmotility due to aganglionosis in the colon, rectum, and possibly the small intestine, which leads to abnormal intestinal motility or obstruction. The presentation of EMG can be similar to that of HD (1).

In a limited number of pediatric patients, the diagnosis of EMG and concomitant HD have been reported (2, 3).

A case of pseudo-obstruction in EMG occurring in a child with HD diagnosis is discussed along with a pediatric literature review.

## Case Presentation

A boy aged 2 years and 6 months presented with refractory constipation and abdominal distension and was admitted to the Pediatric Surgical Unit. He had passed meconium within 24 h of birth in the postnatal ward with no signs of abdominal distension; progressively he developed chronic constipation.

**Figure 1:**
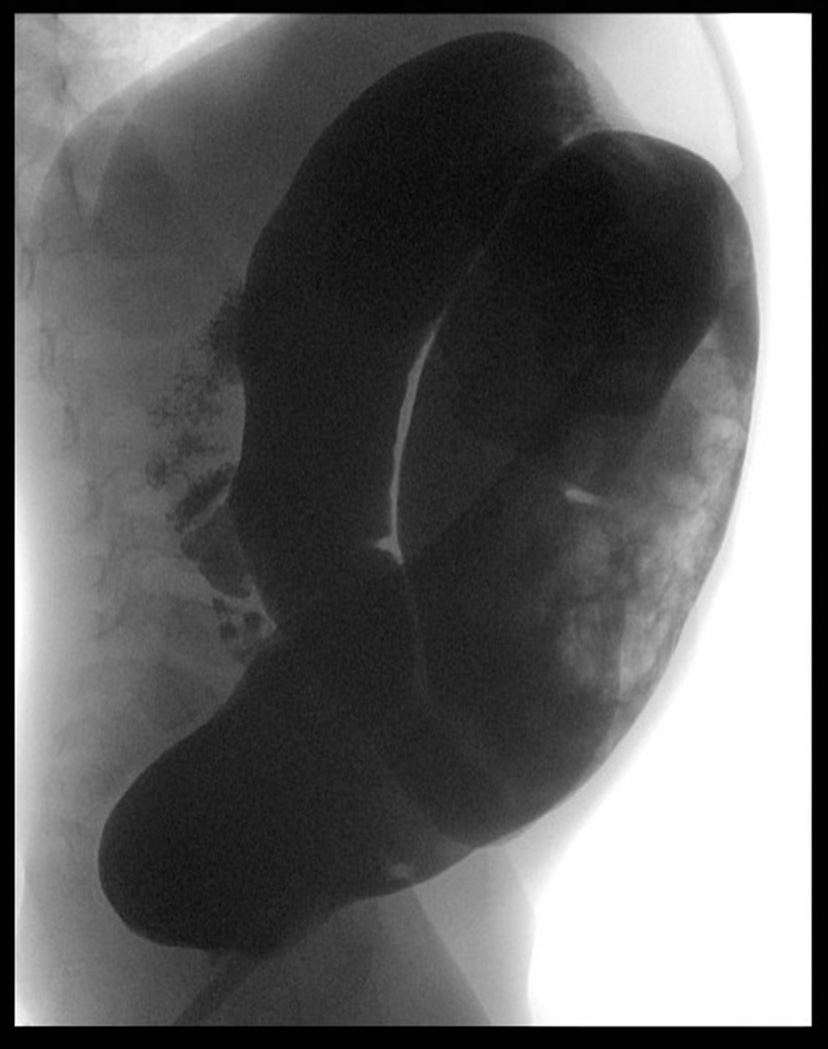
Radiologic evaluation at admission. Barium enema consistent with suspicion OF HD.

At the clinical evaluation, his abdomen was found to be tender and distended, although quiet. Abdominal radiography showed a distal obstruction (Figure 1). Suction rectal biopsies demonstrated nerve hypertrophy. Transanal Soave pullthrough was performed and at the intraoperative evaluation the absence of ganglion cells in the submucosal and myenteric plexus of the rectum and sigmoid colon confirmed the length of HD. The aganglionic colon was resected and the normally innervated bowel was brought down and sutured to the area just above the anal sphincter.

At the age of 3 years and 2 months, an infectious enterocolitis (C. Difficile) confirmed by the finding of the specific toxin by microbiological laboratory testing occurred and an antibiotic treatment was administered, with resolution of symptoms.

**Figure 2:**
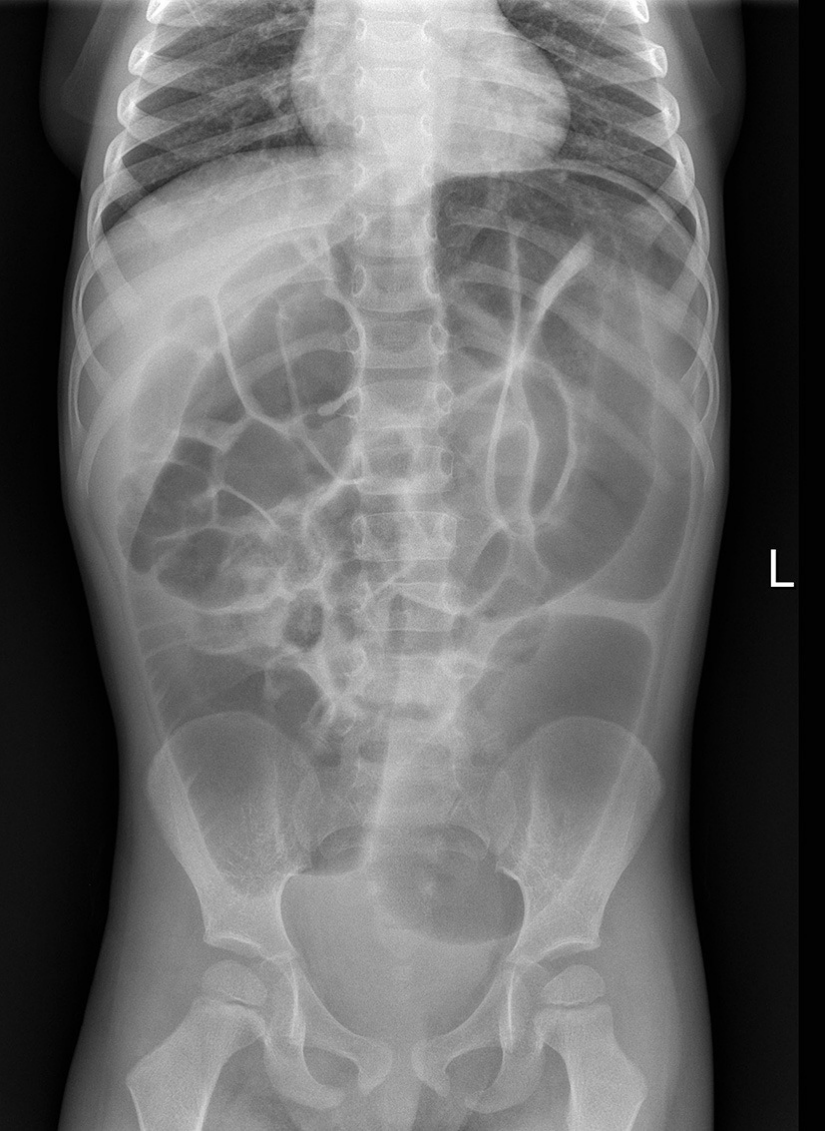
Postoperative plain X ray at the time diagnosis of eosinophilic myenteric gangliositis.

**Figure 3:**
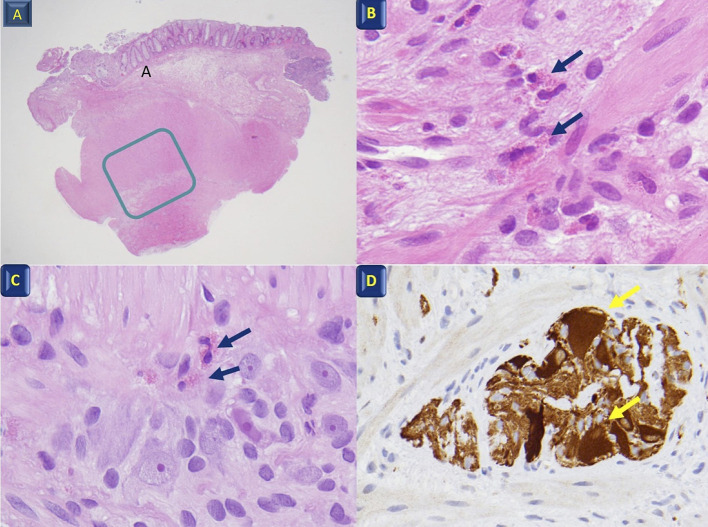
Histological features. **(A)** Full-thickness colon biopsy; the rectangle indicates the muscularis propria and the myenteric Plexus X4; **(B)** Muscularis propria with eosinophil infiltration (arrows) X60; **(C)** Myenteric plexus with eosinophil infiltration (arrows) between the ganglion and glial cells X40; **(D)** Calretinin ganglion cell positivity (arrows) X40.

One month later, he presented with marked abdominal distension and melena; occasional episodes of vomiting was also reported. Abdominal obstruction was detected at radiography (Figure 2). Upper endoscopy and colonoscopy were then performed. Stomach and small bowel mucosal biopsies were normal. Full-thickness surgical distal colon biopsies showed the presence of mature ganglion cells and revealed an eosinophilic-predominant inflammatory infiltrate in the myenteric plexus and surrounding tissues of sigmoid colon and rectum (>60 eosinophils/ 10 HPF, X40) (Figure 3). An EMG was then diagnosed.

The patient had peripheral eosinophilia (8%) and an elevated age-related IgE concentration (423kU/l). A panel of specific IgE tests (immuno-CAP system) was then performed, showing sIgE positive for casein, α-lactalbumin, β-lactoglobulin, egg white, wheat, and maize.

Avoidance diet for cow's milk proteins, egg, wheat and maize was then prescribed. An elemental formula was introduced as a milk substitute; the symptoms resolution was observed.

**Figure 4:**
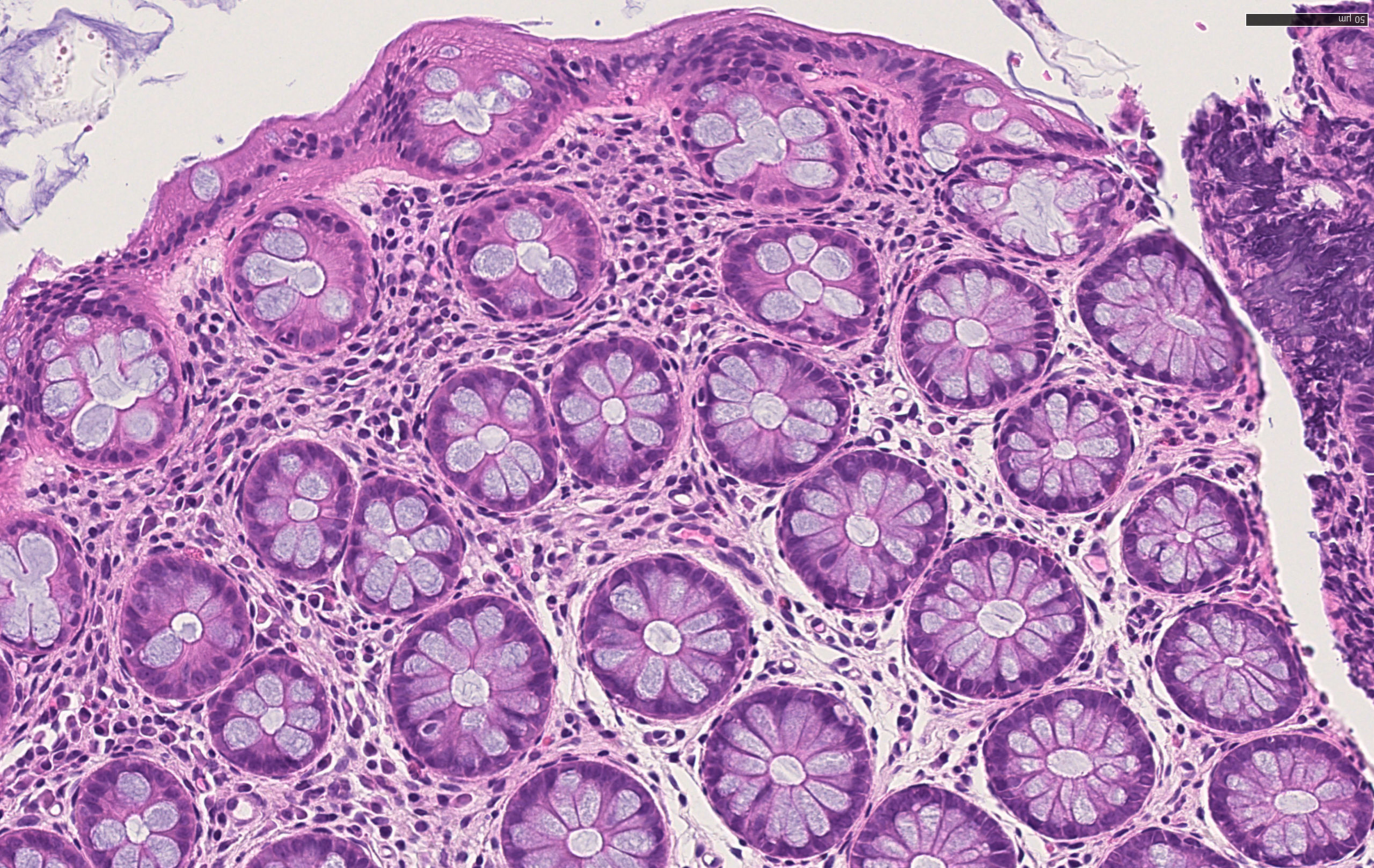
Histological feautures after dietary modification. Colonic mucosa with normal crypts, and rare eosinophils in the lamina propria H&E x40.

After 3 months of dietary modification, colonoscopic mucosal biopsies showed a mild chronic inflammatory cell infiltrate of lymphocytes in rectal tissue, with evidence of histologic remission of intestinal eosinophilic infiltrate (15 HPF in the sigmoid colon, 12 and 10 HPF in the rectum at 10 and 2 cm from anal orifice, respectively) (Figure 4).

At 15 months from the start of the diet, colon biopsies showed a complete resolution of esosinophilic infiltrate and the child's well-being persisted.

## Discussion

Intestinal pseudo-obstruction is characterized by the impairment of gastrointestinal propulsion of the gut content in the absence of obstructive lesions (4). It may occur as a consequence of the failure of the intestinal motility due to a muscular disorder, a neurological disorder or both. Additionally, bowel dysmotility intestinal pseudo-obstructive syndrome may be secondary to EMG (1).

A limited number of cases has been described of different presentations of acquired and reversible functional intestinal obstructive syndrome in association with eosinophilic ganglionitis and eosinophilic colitis in pediatrics. Schappi et al. (1) described three children with severe functional intestinal obstruction and eosinophilic myenteric inflammatory infiltrate, with none of the immunological characteristics of lymphocytic ganglionitis; all responded symptomatically to immunosuppression/anti-inflammatory treatments.

The presence of EMG with concomitant HD is rarely reported in pediatrics. Phillips et al. described a neonate with eosinophilic infiltrates extending into the aganglionic segments of the bowel (2). Lowichik et al. (3) described eosinophilic infiltration in seven children with short-segment HD. We described a novel case of EMD occurring in a pediatric patient affected by HD. Contrary to other cases, in our patient the diagnosis of EMD was not concomitant with HD; it was detected 1 year after HD diagnosis and 1 month after infectious enterocolitis.

The pathogenesis of EMD has yet to be fully elucidated. As reported paraneoplastic syndrome, infection, or primary neurologic disorders have been considered as factors involved in EMG (2); in our case, the pathogenetic role of infection was not excluded. Moreover, given the presence of specific IgE, the importance of allergens could be considered.

EMD diagnosis is made based on symptoms and histological examination results. Two-third of patients had symptoms, mainly diarrhea and abdominal pain, poor feeding, vomiting and more rarely, as in this reported case, a pseudo-intestinal obstruction may occur; (5). Pathognomonic histological features in colon biopsies showed eosinophil density to be greater than normal for site (6).

Surgical resection of the aganglionic intestinal segment and restoration of intestinal continuity is the gold standard for HD treatment. In some patients with HD, postoperative intestinal dysmotility persists, most often manifested as chronic constipation and recurrent episodes of enterocolitis (7). Postoperative enterocolitis remains one of the most feared complications in HD and up todate the management remains a challenge. Our case showed that the recurrence of the intestinal dysmotility post-surgical intervention in HD may be also associated with EMD, which is a particular form of neuronal enteropathy, causing intestinal dysmotility. Dietary modification, immunosuppression, anti-inflammatory medications or a combination of these treatments may be adopted to manage EMG. In some pediatric patients, steroids were necessary for symptoms resolution.

To our knowledge, only one case of EMG which responded to dietary modification alone has been described (2). In contrast to our case, the diagnosis of EMG was made in the first month of age and no associated factors were observed.

In summary, we described the first case of postoperative infectious enterocolitis due to EMG occurring in HD. At the onset, EMG may be associated with postoperative functional intestinal obstruction, secondary to mechanical damage as a consequence of eosinophilic infiltration of the intestinal musculature (8). Dietary modifications alone resulted in adequate symptom relief. Long term follow-up is mandatory to evaluate the child's well-being and the timing of the food reintroduction.

## Data Availability Statement

The raw data supporting the conclusions of this article will be made available by the authors, without undue reservation.

## Ethics Statement

Ethical review and approval was required for the study on human participants in accordance with the local legislation and institutional requirements. Written informed consent to participate in this study was provided by the participants' legal guardian/next of kin.

## Author Contributions

ED'A performed allergological assessment, baseline evaluation and follow up. ED'A and VC wrote and supervised the manuscript with input from all of the authors. MM, LM, and GS performed the surgical interventions. VV and MN performed the histological evaluations and supervised the manuscript. GP supervised the manuscript. All authors discussed the results and approved the final version of the manuscript.

## Conflict of Interest

The authors declare that the research was conducted in the absence of any commercial or financial relationships that could be construed as a potential conflict of interest.
